# Body mass index influences Antimüllerian Hormone and inhibin B in adult males

**DOI:** 10.3389/fendo.2025.1547267

**Published:** 2025-05-20

**Authors:** Wen Zhou, Huanqun Zhou

**Affiliations:** Department of Reproductive Medicine, The Second Affiliated Hospital of Guangzhou University of Chinese Medicine, Guangzhou, China

**Keywords:** BMI, AMH, INHB, NHANES, cross-sectional analysis, non-linear relationship

## Abstract

**Background:**

The interplay between obesity and male reproductive health, particularly concerning reproductive hormone fluctuations, is a well-documented concern. Despite varied findings on the BMI-AMH/INHB relationship, this study utilized NHANES data (1999-2004) to clarify this association, aiming to refine the assessment of obesity’s effects on the reproductive hormone levels of adult male Americans.

**Methods:**

We conducted a cross-sectional analysis involving 728 men aged 20 and older. Height and weight were measured by trained staff, and hormone levels were determined using the ELISA method. We performed weighted multiple linear regression to assess the associations between BMI and AMH/INHB, including subgroup interactions, and utilized smoothing curve fitting to analyze nonlinear relationships, along with a threshold effect analysis to evaluate key thresholds.

**Results:**

Participants in higher BMI quartiles showed a declining trend in AMH levels (P=0.16) and a significant reduction in INHB levels (P<0.01). A negative correlation between BMI and AMH (β: -0.15, 95%CI: -0.23 to -0.06, P<0.01) and INHB levels (β: -2.14, 95%CI: -2.98 to -1.31, *P*<0.0001) was observed, with these correlations remaining statistically significant (AMH: β: -0.12, 95%CI: -0.23 to -0.01, *P*<0.05; INHB: β: -1.50, 95%CI: -2.66 to -0.34, *P*<0.05) after adjusting for relevant confounders. However, the effect size for AMH was relatively low, which may limit its clinical significance. In the fully adjusted model, the increase in BMI in Q4 was linked to decreases of 1.62 ng ml-1 in AMH and 18.20 pg ml^-1^ in INHB, but these associations were not statistically significant (P>0.05). The association between BMI and AMH/INHB showed no significant interaction effects across all covariates (P>0.05 for the interaction), although negative correlations were present in most subgroups (P<0.05). While both AMH and INHB declined with increasing BMI, they displayed nonlinear relationship at key thresholds of 30.78 kg m-² (below: β=0.02, P>0.05; above: β=-0.30, P<0.05) and 33.86 kg m^-^² (below: β=-1.24, P=0.05; above: β=-3.22, P<0.05).

**Conclusions:**

BMI is associated with a relatively independent negative correlation with serum AMH and INHB levels in adult men, which is more noticeable in obese men and shows no significant interaction with other confounding factors. However, due to the low effect size of BMI/AMH, caution is needed in interpreting its clinical significance. Although we found a non-linear relationship and key thresholds between these variables, further studies with larger sample sizes are needed to confirm these findings.

## 1 Introduction

Since 1990, the prevalence of obesity among adults worldwide has more than doubled, underscoring a significant global health concern (1); particularly, it relates to male infertility, as obesity may increase the risk of male infertility by affecting hormone levels, sperm quality, and reproductive function (2).

Anti-Müllerian Hormone (AMH), secreted by Sertoli cells in the testes, is crucial for male reproductive development, particularly in embryonic sexual differentiation and testicular development during puberty (3). Inhibin B (INHB), a glycoprotein hormone secreted by Sertoli cells, inhibits the synthesis and secretion of follicle-stimulating hormone (FSH) from the pituitary gland through a negative feedback mechanism. As FSH collaborates with testosterone to regulate spermatogenesis, INHB is considered an important biomarker for this process (4).

Despite previous investigations into the relationship between BMI and levels of AMH and INHB, findings remain inconsistent. In severely obese men, BMI negatively correlates with semen quality and reproductive hormone levels, while weight loss is significantly associated with increased AMH levels, suggesting that weight reduction may enhance reproductive health (5). In addition, among healthy expectant fathers, AMH levels decrease with increasing BMI and exhibit complex relationships with reproductive hormones such as FSH and luteinizing hormone (LH), indicating that AMH may serve as a critical biomarker for male reproductive health (6). Although these studies show a close relationship between BMI and AMH, their focus on severely obese individuals or healthy expectant fathers may limit the generalizability of the findings. Several studies suggest that serum INHB levels may decrease with increasing BMI, indicating a potential impact of obesity on male reproductive potential (7, 8). However, these findings are largely derived from specific populations, such as young males or particular ethnic groups, and do not establish a clear trend association.

Furthermore, while some studies indicate that higher BMI correlates with decreased levels of AMH and INHB, others propose that this relationship may be influenced by factors such as age, lifestyle, health parameters, and sex hormone levels. These discrepancies hinder a comprehensive understanding of the effects of obesity on male reproductive hormones. This study aims to clarify the association between BMI and the levels of AMH and INHB in American adult males by utilizing publicly available data from the National Health and Nutrition Examination Survey (NHANES) conducted between 1999 and 2004. By leveraging extensive datasets, we seek to control for potential confounding variables, thereby providing a more accurate evaluation of BMI’s impact on these reproductive hormones. The anticipated findings are expected to enhance our understanding of the effects of obesity on male reproductive health.

## 2 Methods

### 2.1 Study population

**Figure 1:**
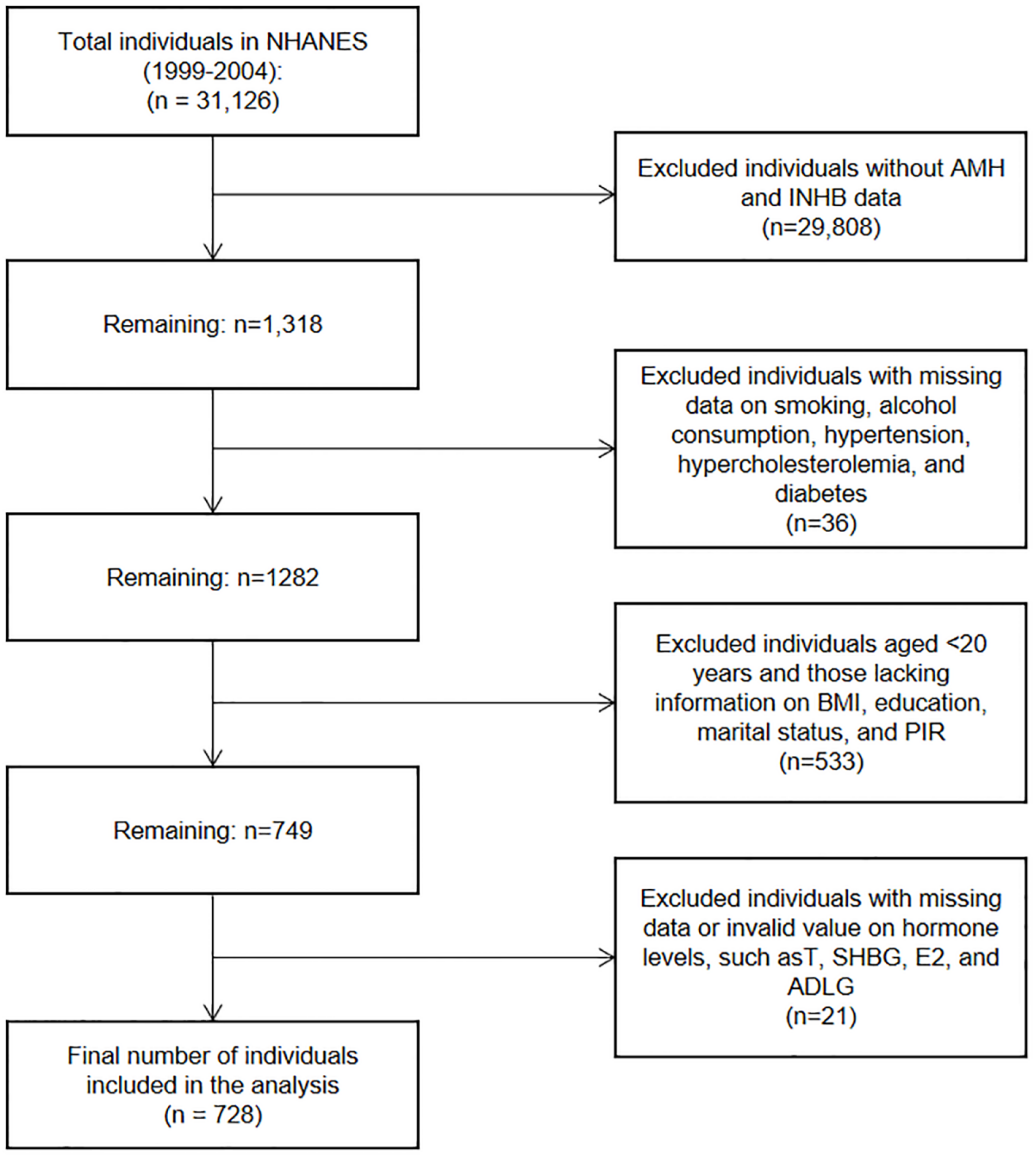
Flowchart of participant selection in the NHANES study (1999-2004).

This cross-sectional study investigated the relationship between BMI and serum levels of AMH and INHB in men. Data were sourced from the NHANES, a program conducted by the National Center for Health Statistics in the United States. We analyzed data from three NHANES cycles spanning from 1999 to 2004, focusing on male participants aged 20 years or older who had complete serum AMH and INHB data. As shown in **Figure 1**, a total of 728 participants were included in the analysis. Additional information was collected on various factors, including demographic information, smoking status, alcohol consumption, hypertension, hyperlipidemia, diabetes and sex hormone levels (Testosterone (T), Sex Hormone Binding Globulin (SHBG), Estradiol (E2), Androstanedione glucuronide (ADLG)).

### 2.2 Measurement of BMI

Trained examiners measured height and weight at mobile examination centers (MEC) during the NHANES survey. Each measurement was taken twice, with a third measurement performed if the difference exceeded 0.5 kg. BMI was calculated using the formula: BMI = Weight (kg)/Height (m)².

### 2.3 Serum AMH and INHB measurement

Serum AMH was measured using the Beckman Coulter second-generation AMH ELISA kit. INHB levels were assessed via ELISA following sodium dodecyl sulfate pretreatment and heating to enhance specificity. Color intensity was measured at 490 nm, with a sensitivity of <15 pg ml^-1^ and minimal cross-reactivity for inhibin A. AMH is reported in ng ml^-1^, while INHB is reported in pg ml^-1^.

### 2.4 Confounding variables

The confounding variables included age, race, education, marital status, family poverty income ratio (PIR), smoking status, alcohol use, hypertension, hypercholesterolemia, diabetes, T, SHBG, ADLG, and E2 levels. Age was categorized into three groups: <35 years, 35–64 years, or ≥65 years. Racial categories included Mexican American, other Hispanic, non-Hispanic White, non-Hispanic Black, and other or multiple races. Education was classified into three levels: less than high school, high school or equivalent, and above high school. PIR were categorized as <1.30, 1.30-3.49, and ≥3.5. Smokers were defined as individuals with a lifetime history of smoking more than 100 cigarettes, and drinkers were those who had consumed at least 12 alcoholic beverages in their lifetime or the past year. Hypertension was defined as a prior diagnosis, use of antihypertensive medications, or blood pressure readings of systolic ≥140 mm Hg or diastolic ≥90 mm Hg. Hypercholesterolemia was classified by serum cholesterol levels of ≥240 mg dl^-1^, a previous diagnosis of high cholesterol, or the use of lipid-lowering medications. Diabetes was diagnosed based on medical history or fasting blood glucose levels of ≥126 mg dl^-1^. The levels of T, SHBG, ADLG, and E2 were classified using tertiles in subgroup analyses.

### 2.5 Statistical analysis

In this study, statistical analyses were conducted using Empower version 4.2 (X&Y Solutions, Inc., Boston, MA, United States) and R version 4.2.0 (R Foundation). We used NHANES subsample weights to ensure that the results accurately represent the U.S. population. New weights were calculated as 2/3*WTSAF4YR+1/3*WTSAF2YR, due to the special periods of 1999–2000 and 2001-2002, based on the fasting blood glucose dataset. Descriptive statistics were reported by BMI quartiles, with continuous variables presented as Median (IQR) and categorical variables expressed as percentages (95%CI). Statistical analyses included weighted kruskal-wallis H test and weighted chi-square test. Weighted multiple linear regression models were used to assess the relationships between BMI and AMH and INHB across three models: Model 1 (unadjusted), Model 2 (adjusted for age, race, education, PIR, and marital status), and Model 3 (further adjusted for smoking, alcohol consumption, hypertension, hyperlipidemia, diabetes, T, SHBG, ADLG, and E2). Adjusted regression coefficients (Beta coefficients) and 95%CI were reported for each model. Subgroup analyses evaluated differences in associations among various groups while controlling for other variables. Additionally, generalized additive models (GAM) were employed for smooth curve fitting analysis to explore non-linear relationships between BMI and AMH and INHB levels, adjusting for all confounding factors. Furthermore, threshold effect analysis was conducted to identify valuable inflection points.

## 3 Results

### 3.1 Demographic and clinical characteristics

As shown in **Table 1**, AMH levels tended to decline with increasing BMI (*P*=0.16), while INHB, T, and SHBG levels were significantly lower in higher BMI quartiles (*P<0.01*). Participants in the higher BMI quartiles exhibited a notable increase in E2 levels, age, the marriage or cohabitation status, and prevalence of hypertension and diabetes (*P*<0.01). No significant differences were observed in ADLG levels, PIR, racial categories, education level, smoking status, alcohol consumption, and hypercholesterolemia prevalence across BMI quartiles (*P*>0.05).

**Table 1:** Weighted analysis of demographic and clinical characteristics by BMI quartiles.

Characteristics	BMI Quartiles (kg m^-2^)				*P*
	Q1 (16.01-24.55)	Q2 (24.56-27.25)	Q3 (27.27-30.48)	Q4 (30.50-62.99)	
AMH (ng ml^-1^)	7.43 (4.31 ,14.22)	7.09 (3.54 ,11.05)	6.19 (3.71 ,10.09)	5.47 (3.65 ,10.10)	0.16
INHB (pg ml^-1^)	138.40 (102.22 ,183.94)	122.63 (88.34 ,166.40)	125.54 (89.10 ,179.43)	106.24 (79.91 ,139.98)	<0.01
T (ng ml^-1^)	6.24 (5.08 ,7.85)	5.12 (4.27 ,6.37)	4.19 (3.34 ,5.20)	4.08 (2.86 ,5.26)	<0.0001
SHBG (nmol l^-1^)	40.69 (30.40 ,54.80)	34.14 (26.90 ,43.13)	28.06 (19.43 ,39.91)	26.44 (17.72 ,38.02)	<0.0001
E2 (pg ml^-1^)	30.44 (23.93 ,43.81)	27.02 (19.58 ,37.94)	26.90 (20.18 ,35.65)	32.26 (25.61 ,45.10)	0.01
ADLG (ng ml^-1^)	6.72 (4.84 ,9.06)	7.41 (5.20 ,10.17)	6.69 (5.21 ,9.08)	7.64 (5.95 ,10.67)	0.11
Age (years)	38.00 (29.00 ,50.00)	44.00 (32.00 ,53.00)	45.00 (32.00 ,57.00)	44.00 (32.00 ,56.00)	0.02
PIR (%)	2.82 (1.40 ,5.00)	3.09 (1.73 ,5.00)	3.59 (1.78 ,5.00)	3.35 (1.82 ,5.00)	0.33
BMI (kg m^-2^)	22.14 (20.73 ,23.67)	25.87 (25.06 ,26.46)	28.99 (28.15 ,29.74)	34.17 (32.07 ,36.43)	<0.0001
Race (%)					0.61
Mexican American	6.35 (3.82 ,10.36)	8.06 (5.20 ,12.28)	8.20 (5.13 ,12.86)	6.93 (3.88 ,12.08)	
Other Hispanic	3.51 (1.17 ,10.08)	3.03 (0.92 ,9.51)	5.48 (2.28 ,12.58)	5.39 (2.10 ,13.18)	
Non-Hispanic White	67.11 (57.82 ,75.23)	74.40 (64.77 ,82.12)	74.60 (66.45 ,81.32)	71.72 (63.30 ,78.85)	
Non-Hispanic Black	14.56 (9.51 ,21.64)	9.38 (5.84 ,14.74)	7.43 (4.72 ,11.50)	9.56 (6.49 ,13.88)	
Other Race	8.48 (4.48 ,15.46)	5.13 (2.03 ,12.40)	4.30 (1.55 ,11.42)	6.39 (3.09 ,12.75)	
Education level (%)					0.71
Less than high school	18.35 (13.60 ,24.29)	22.21 (15.80 ,30.29)	16.70 (10.92 ,24.71)	20.17 (14.35 ,27.60)	
High school or ged	20.93 (12.70 ,32.53)	22.13 (15.35 ,30.83)	29.00 (21.12 ,38.39)	24.44 (18.13 ,32.08)	
Above high school	60.72 (49.42 ,70.97)	55.66 (45.54 ,65.32)	54.30 (45.77 ,62.57)	55.39 (45.74 ,64.65)	
Marital status (%)					<0.01
Married or living with partner	58.93 (49.29 ,67.93)	69.27 (62.29 ,75.47)	76.29 (65.75 ,84.36)	78.31 (69.64 ,85.04)	
Living alone	41.07 (32.07 ,50.71)	30.73 (24.53 ,37.71)	23.71 (15.64 ,34.25)	21.69 (14.96 ,30.36)	
Smoking status (%)					0.11
Yes	62.33 (53.59 ,70.33)	59.62 (48.81 ,69.58)	54.92 (44.79 ,64.65)	47.64 (41.26 ,54.09)	
No	37.67 (29.67 ,46.41)	40.38 (30.42 ,51.19)	45.08 (35.35 ,55.21)	52.36 (45.91 ,58.74)	
Alcohol consumption (%)					0.05
Yes	93.10 (88.20 ,96.06)	98.26 (93.58 ,99.55)	94.39 (89.01 ,97.21)	88.66 (75.58 ,95.18)	
No	6.90 (3.94 ,11.80)	1.74 (0.45 ,6.42)	5.61 (2.79 ,10.99)	11.34 (4.82 ,24.42)	
Hypertension (%)					<0.0001
Yes	19.53 (13.60 ,27.23)	29.64 (23.67 ,36.39)	35.22 (26.28 ,45.34)	50.66 (41.35 ,59.92)	
No	80.47 (72.77 ,86.40)	70.36 (63.61 ,76.33)	64.78 (54.66 ,73.72)	49.34 (40.08 ,58.65)	
Hypercholesterolemia (%)					0.10
Yes	27.03 (20.91 ,34.17)	38.37 (30.03 ,47.44)	34.80 (25.70 ,45.15)	40.81 (33.39 ,48.66)	
No	72.97 (65.83 ,79.09)	61.63 (52.56 ,69.97)	65.20 (54.85 ,74.30)	59.19 (51.34 ,66.61)	
Diabetes (%)					<0.0001
Yes	2.34 (0.92 ,5.79)	7.87 (4.01 ,14.84)	10.86 (6.39 ,17.86)	19.90 (13.18 ,28.91)	
No	97.66 (94.21 ,99.08)	92.13 (85.16 ,95.99)	89.14 (82.14 ,93.61)	80.10 (71.09 ,86.82)	

Continuous variables were presented as median (IQR) since they did not follow a normal distribution, with *P* values obtained from the weighted Kruskal-Wallis H test. Categorical variables were reported as percentages (95%CI) with *P* values from the weighted Chi-square test.

### 3.2 The association of BMI with serum levels of AMH and INHB

As shown in **Table 2**, Model 1 (unadjusted) indicated negative correlations between BMI and AMH (β: -0.15, 95%CI: -0.23 to -0.06, *P*<0.01) as well as INHB (β: -2.14, 95%CI: -2.98 to -1.31, *P*<0.0001). In Model 3, after adjusting for age, race, education level, marital status, PIR, smoking status, alcohol consumption, hypertension, hypercholesterolemia, diabetes, T, SHBG, ADLG, and E2, these associations remained statistically significant (AMH: β: -0.12, 95%CI: -0.23 to -0.01, *P*<0.05; INHB: β: -1.50, 95%CI: -2.66 to -0.34, *P*<0.05), indicating that each unit increase in BMI is associated with a decrease of 0.12 ng ml^-1^ in AMH levels and 1.50 pg ml^-1^ in INHB levels. However, the effect size for AMH was relatively low, which may limit its clinical significance. Compared to Q1, increases in BMI in Q4 were associated with declines in AMH (β: -1.62, 95%CI: -3.87 to 0.63, *P*>0.05) and INHB (β= -18.20, 95%CI: -38.84 to 2.44, *P*>0.05), but neither result was statistically significant.

**Table 2:** Weighted multivariable linear regression analysis of the association between BMI and serum levels of AMH and INHB.

Variables	β (95%CI) *P*-value		
	Model 1	Model 2	Model 3
BMI vs. AMH			
Continuous	-0.15 (-0.23, -0.06) <0.01	-0.11 (-0.19, -0.03) 0.02	-0.12 (-0.23, -0.01) 0.03
Q1**(**16.01-24.52**)**	Ref.	Ref.	Ref.
Q2**(**24.55-27.19**)**	-1.28 (-3.72, 1.16) 0.31	-0.78 (-3.02, 1.46) 0.50	-1.06 (-3.40, 1.27) 0.34
Q3**(**27.21-30.50**)**	-1.53 (3.52, 0.46) 0.14	-0.54 (-2.35, 1.28) 0.57	-0.85 (-2.90, 1.20) 0.39
Q4**(**30.51-62.99**)**	-2.28 (-4.28, -0.29) 0.03	-1.66 (-3.44, 0.12) 0.08	-1.62 (-3.87, 0.63) 0.14
P for trend	0.03	0.11	0.20
BMI vs. INHB			
Continuous	-2.14 (-2.98, -1.31) <0.0001	-2.11 (-2.96, -1.26) <0.0001	-1.50 (-2.66, -0.34) 0.01
Q1**(**16.01-24.52**)**	Ref.	Ref.	Ref.
Q2**(**24.55-27.19**)**	-16.72 (-28.21, -5.24) <0.01	-16.85 (-28.74, -4.96) 0.01	-13.11 (-27.87, 1.63) 0.08
Q3**(**27.21-30.50**)**	-14.17 (-30.27, 1.93) 0.09	-11.61 (-28.24, 5.01) 0.18	-4.74 (-24.94, 15.46) 0.62
Q4**(**30.51-62.99**)**	-30.93 (-46.41, -15.46) <0.001	-29.69 (-44.93, -14.45) <0.001	-18.20 (-38.84, 2.44) 0.08
P for trend	<0.01	<0.01	0.17

Model 1: Unadjusted.

Model 2: Adjusted for age, race, education level, marital status, and PIR.

Model 3: Adjusted for age, race, education level, marital status, PIR, smoking status, hypertension, hypercholesterolemia, alcohol consumption, diabetes, T, SHBG, ADLG, and E2.

Statistical analysis: Weighted multivariable linear regression analysis was performed to estimate survey-weighted coefficients (95%CI) and *P* values for AMH and INHB.

### 3.3 Subgroup analyses and interaction effects

**Figure 2:**
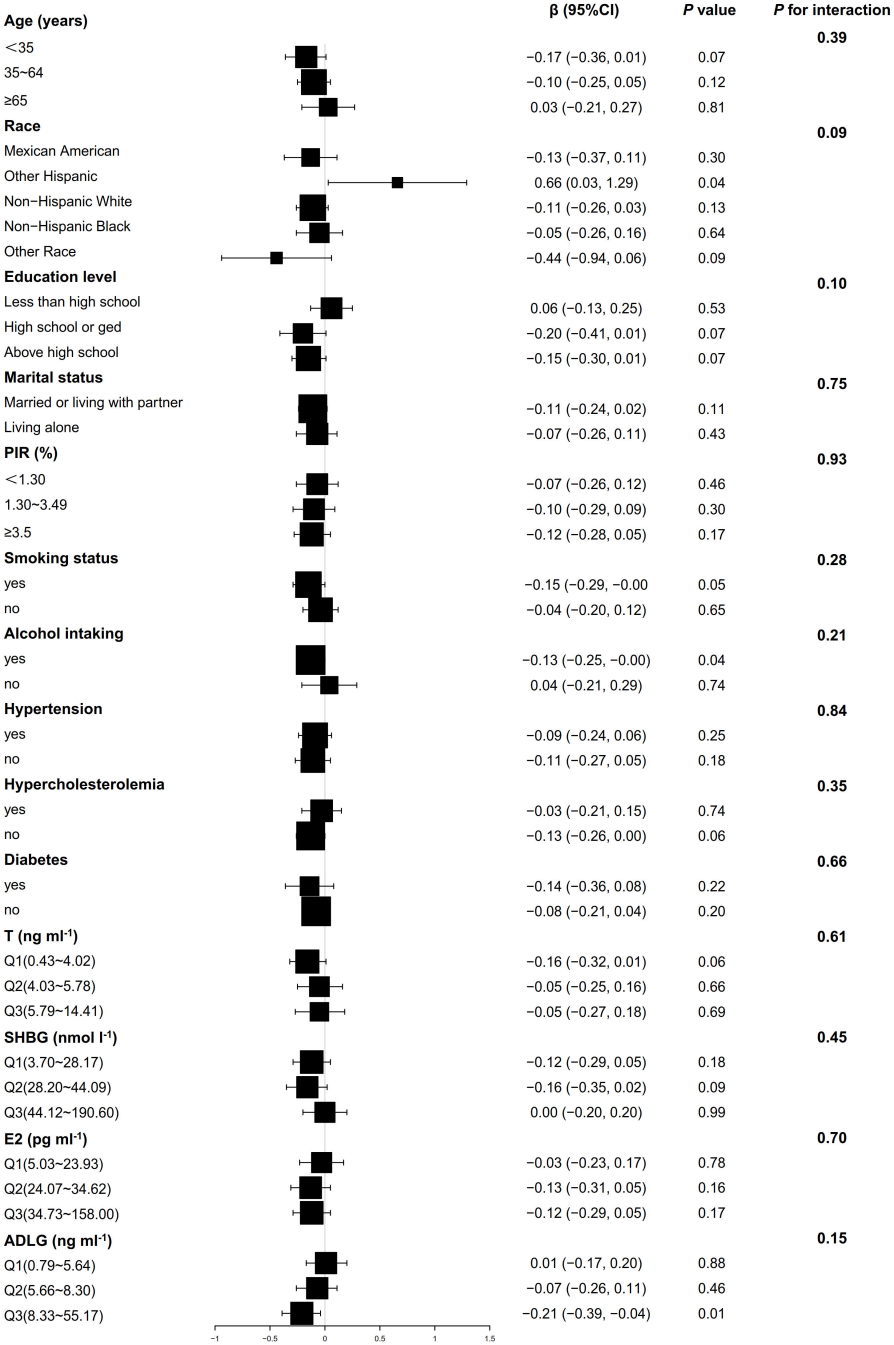
Weighted multivariable linear regression analysis of subgroup interaction effects on the association between BMI and AMH: A forest plot. Model: Adjusted for all potential confounders excluding self-referential factors, results are presented as β, 95% CI, and P-values. Forest plot: Squares denote coefficients for each subgroup, with horizontal lines indicating 95% CI. P for interaction: This statistic assesses the significance of interaction effects among subgroups.

**Figure 3:**
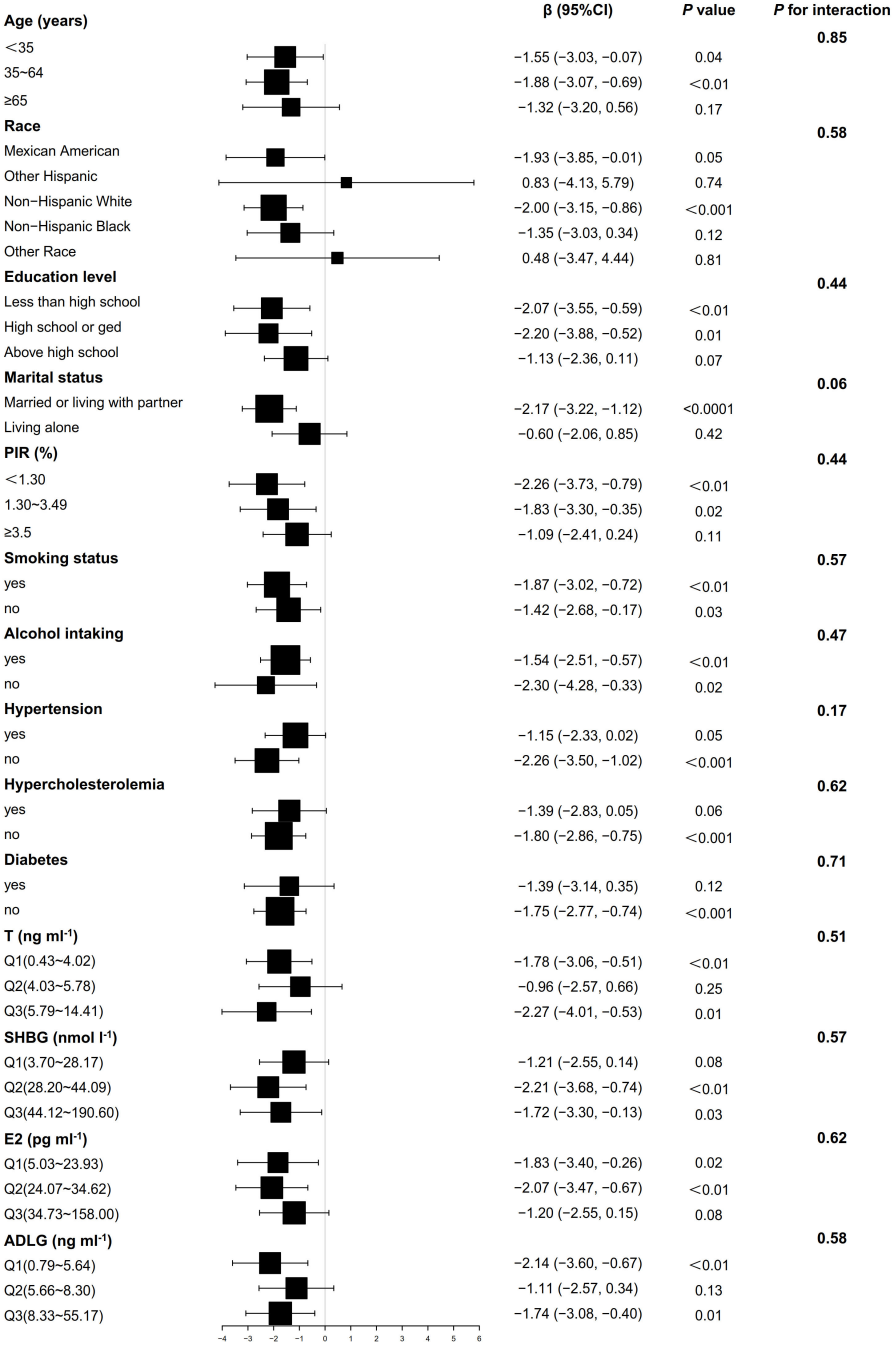
Weighted multivariable linear regression analysis of subgroup interaction effects on the association between BMI and INHB: A forest plot. Model: Adjusted for all potential confounders excluding self-referential factors, results are presented as β, 95% CI, and P-values. Forest plot: Squares denote coefficients for each subgroup, with horizontal lines indicating 95% CI. P for interaction: This statistic assesses the significance of interaction effects among subgroups.

**Figure 2** shows that the association between BMI and AMH did not exhibit significant interaction effects across all covariate subgroups (*P*>0.05). In the smoking, drinking, and high ADLG groups, BMI was significantly negatively correlated with AMH (*P*<0.05), while it was positively correlated in the other Hispanic group (*P*<0.05). As shown in **Figure 3**, no significant interaction effects were observed between BMI and INHB in various covariate subgroups (*P*>0.05). However, significant negative correlations were found in the following subgroups (*P*<0.05): age (<65 years), Non-Hispanic White, education level (high school or below), marital status (married or cohabiting), PIR<3.50, smoking status (yes or no), alcohol consumption (yes or no), hypertension (yes or no), hyperlipidemia (absent), diabetes (absent), testosterone levels (≤4.02 or ≥5.78 ng ml^-1^), SHBG (≥28.2 nmol l^-1^), E2 (≤34.62 pg ml^-1^), and ADLG (≤5.64 or ≥8.33 ng ml^-1^). Negative correlations were also observed in most other subgroups, although they did not reach statistical significance (*P*>0.05).

### 3.4 Smooth curve fitting analysis and threshold effect analysis

**Figure 4:**
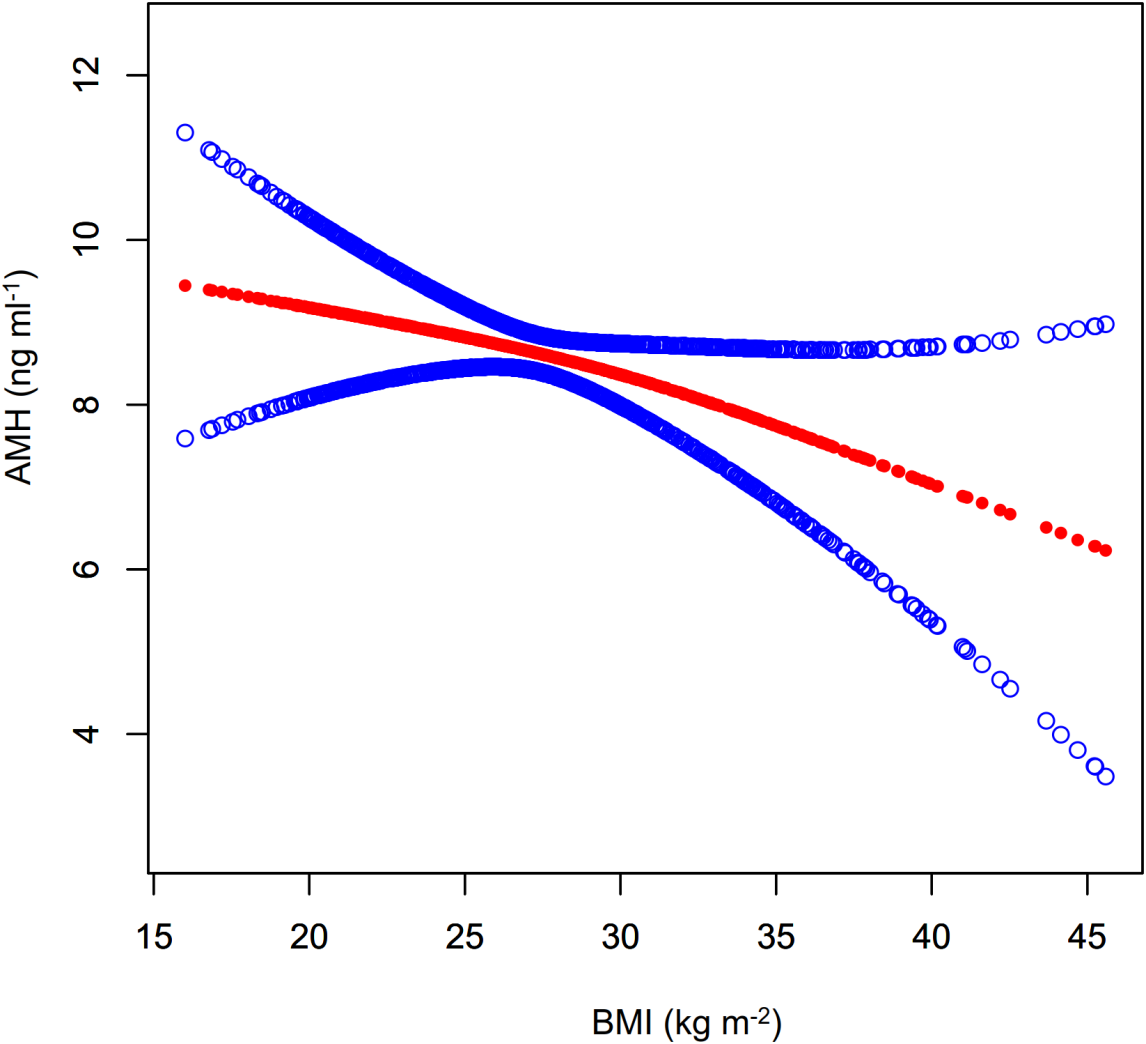
Fitted curve of AMH levels vs. BMI. Model: Adjusted for age, race, education level, marital status, PIR, smoking status, hypertension, hypercholesterolemia, alcohol consumption, diabetes, T, SHBG, ADLG, and E2. Statistical analysis: Preliminary curve fitting using the Generalized Additive Model identified four outliers (BMI/AMH: 49.23/17.00, 49.73/5.74, 57.5/3.37, 62.99/2.49), which were removed. The updated curve fitting shows AMH level trends by BMI (red line) and the 95% CI (blue line).

**Figure 5:**
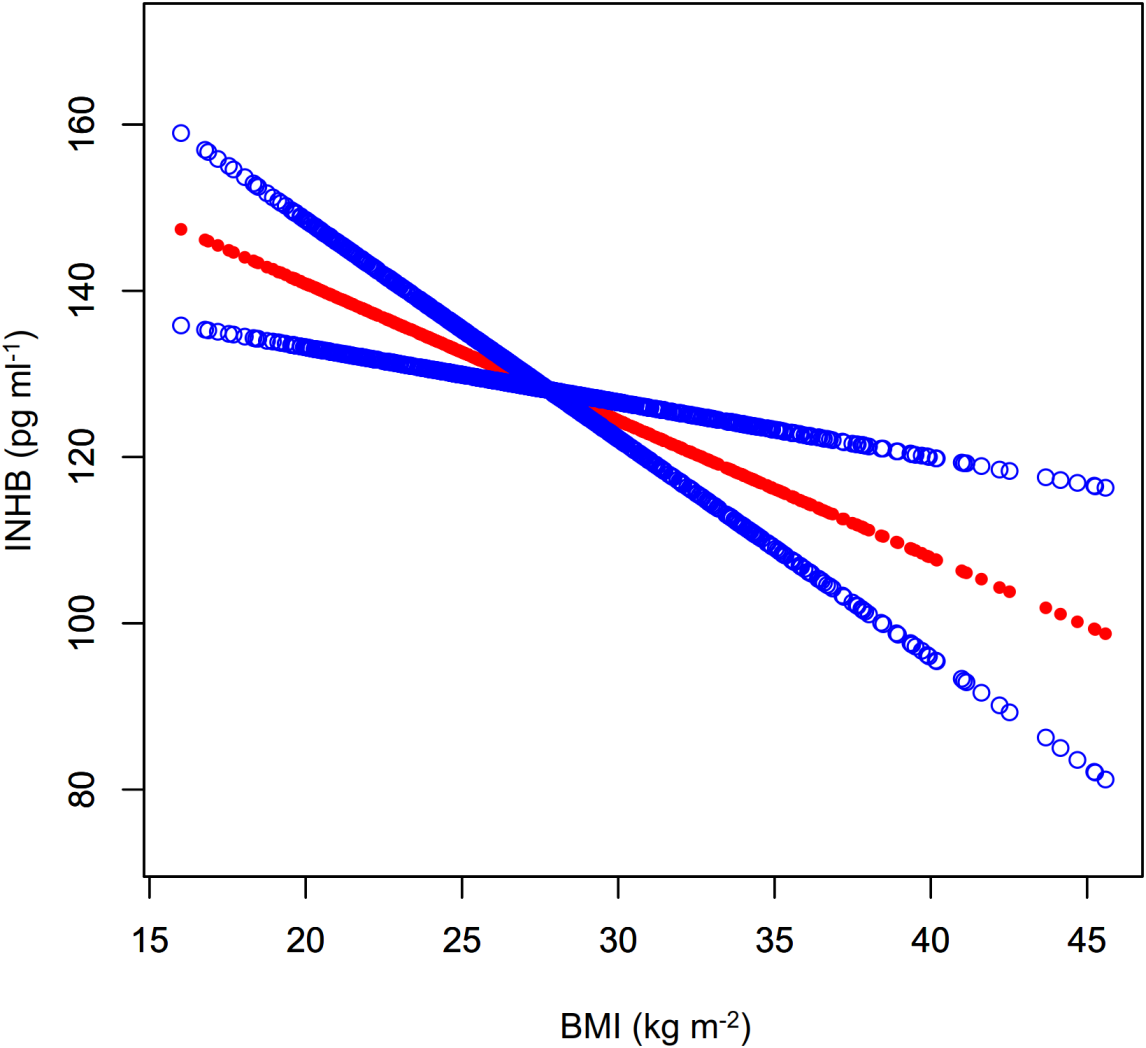
Fitted curve of INHB levels vs. BMI. Model: Adjusted for age, race, education level, marital status, PIR, smoking status, hypertension, hypercholesterolemia, alcohol consumption, diabetes, T, SHBG, ADLG, and E2. Statistical analysis: Preliminary curve fitting using the Generalized Additive Model identified four outliers (BMI/INHBIN:49.23/98.28、49.73/89.41、57.5/118.66、62.99/43.91), which were removed. The updated curve fitting shows INHB level trends by BMI (red line) and the 95% CI (blue line).

As shown in **Figure 4**; **Table 3**, a nonlinear relationship was observed between BMI and AMH levels, with non-significant variation in AMH when BMI was less than 30.78 kg m^-^² (β= 0.02, 95%CI: -0.18 to 0.21, *P*>0.05) and a statistically significant decline when BMI was greater (β= -0.30, 95%CI: -0.56 to -0.03, *P*<0.05). However, the small effect size may impact its clinical significance. Additionally, a significant negative correlation between BMI and INHB levels was found in model I (β= -1.65, 95%CI: -2.63 to -0.66, *P*<0.01) (**Figure 5**; **Table 3**). In model 2, segmented regression analysis identified a threshold at 33.86 kg m^-^², where a negative correlation between BMI and INHB exists below this value, though with a low effect size (β= -1.24, 95%CI: -2.49 to 0.00, *P*=0.05). Above 33.86, each unit increase in BMI results in a significant decrease of 3.22 units in INHB (β= -3.22, 95%CI: -6.33 to -0.10, *P*<0.05) (**Table 3**).

**Table 3:** Threshold effect analysis of the association between BMI and serum levels of AMH and INHB.

Effect Estimates	AMH (ng ml^-1^)	INHB (pg ml^-1^)
Model I		
One linear effect	-0.10 (-0.23, 0.02) 0.10	-1.65 (-2.63, -0.66) <0.01
Model II		
Threshold (K)	30.78	33.86
< K Section Effect 1	0.02 (-0.18, 0.21) 0.86	-1.24 (-2.49, 0.00) 0.05
> K Section Effect 2	-0.30 (-0.56, -0.03) 0.03	-3.22 (-6.33, -0.10) 0.04
Effect difference between 2 and 1	-0.31 (-0.69, 0.06) 0.10	-1.97 (-5.69, 1.74) 0.30
Predicted values at the breakpoint	8.35 (7.36, 9.35)	115.56 (106.56, 124.57)
Likelihood ratio test	0.10	0.29

Model: Adjusted for age, race, education level, marital status, PIR, smoking status, hypertension, hypercholesterolemia, alcohol consumption, diabetes, T, SHBG, ADLG, and E2.

Statistical analysis: Multivariable linear regression analysis was performed to estimate β (95%CI) and p-values for AMH and INHB.

## 4 Discussion

Our study utilized a weighted analysis of NHANES data to examine the impact of BMI on serum levels of AMH and inhibin B in adult men. The results indicated a relatively independent negative correlation between BMI and both AMH and inhibin B levels, with no significant interaction with interested covariates. Further analysis showed that this negative correlation was nonlinear, becoming more pronounced at key BMI thresholds of 30.78 kg m^-^² and 33.86 kg m^-^². Although the negative correlation between BMI and AMH was statistically significant, the small effect size may limit its clinical relevance.

Hadlow et al. (6) studied the relationship between AMH, metabolic parameters, and reproductive hormones in 485 men seeking fatherhood, finding that AMH is negatively correlated with age and BMI, while the correlation between AMH and FSH remains independent of these factors. They also observed a moderate positive correlation between AMH and testosterone, moderated by BMI. Similarly, Andersen et al. (9) found a significant negative correlation between BMI and male sperm parameters, T, INHB, and AMH, underscoring the adverse effects of obesity on reproductive health. In a weight loss intervention study, Håkonsen et al. (5) indicated that reductions in body weight were significantly correlated with increases in total sperm count, semen volume, testosterone, SHBG, and AMH, highlighting the adverse effects of obesity on male reproductive health. My study corroborates these findings by revealing a negative correlation between BMI and AMH and INHB after accounting for most confounding factors, also emphasizing a new finding of a nonlinear relationship at specific BMI thresholds, which may be attributed to differences in study populations. Additionally, Pietiläinen et al. (10) conducted a study involving 64 pairs of healthy male twins, finding that serum AMH levels in post-pubertal men were significantly influenced by genetic factors and negatively correlated with BMI and body fat. However, no genetic correlation between BMI and AMH was identified, suggesting that BMI may affect male reproductive hormone levels through pathways independent of genetic influences. Jensen et al. (7) conducted a study involving 1,558 Danish men and found that serum INHB levels decline as BMI increases. Men with a BMI less than 20 kg m^-^² had significantly higher INHB levels than those with normal or higher BMIs, and declines in INHB were associated with reductions in sperm concentration and total sperm count. Our results differed from theirs, indicating that a BMI around 34 kg m^-^² served as a turning point in influencing AMH and INHB levels, characterized by a gentle slope before this point and an increasing negative slope afterward. This may be due to differences in statistical methods, as curve fitting could have better revealed overall trends. Additionally, their study focused on younger males, while our sample better represented the overall adult male population. However, due to the limitations of NHANES, while we included various potential confounding factors such as demographics, health status, lifestyle habits, T, SHBG, E2, and ADLG, we were still unable to address all factors, such as FSH and physical activity, which may have influenced our statistical results.

AMH plays a certain role in the development of the male reproductive system. During embryonic development, AMH is secreted by Sertoli cells in the testes, activating the Smad signaling pathway through the AMHRII receptor, which leads to the regression of the Müllerian ducts and promotes male differentiation (3, 11, 12). In prepubertal males, AMH levels can serve as an indicator of Sertoli cell activity, providing a reference for assessing testicular development and the progression of puberty (13). However, the regulatory role of AMH in spermatogenesis (14) and its interactions with sex hormones (15) remain subjects of controversy. The negative correlation between obesity and male AMH levels may be driven by multiple mechanisms. First, obese men often exhibit hypogonadism, characterized by decreased T and elevated E2 levels, which may inhibit the hypothalamic-pituitary-gonadal (HPT) axis, thereby reducing the secretion of FSH and indirectly affecting the secretion of AMH by Sertoli cells (16). Second, chronic low-grade inflammation can impair the testicular microenvironment by increasing cytokines (such as IL-6 and TNF-α), inhibiting the function of Sertoli cells (17). Additionally, insulin resistance and visceral fat accumulation may further disrupt Sertoli cell function and alter the expression of AMH-related genes through epigenetic mechanisms like DNA methylation.17 However, much of the evidence supporting these mechanisms is derived from animal studies, and there is still insufficient human data; thus, these findings should be interpreted with caution.

INHB is a glycoprotein hormone secreted by Sertoli cells in the testes, and it regulates the synthesis and secretion of FSH through a negative feedback mechanism (4, 18, 19). The serum level of INHB directly reflects the spermatogenic function of the testes and the activity of Sertoli cells, making it a key biomarker for assessing male reproductive function. Obesity may lead to dysfunction of the HPT axis, resulting in reduced secretion of LH and consequently decreased T synthesis, which indirectly affects Sertoli cell function (20). Additionally, obesity is often associated with insulin resistance, leading to lowered levels of SHBG and reduced free T, further impairing Sertoli cell function (21). Moreover, elevated levels of inflammatory and adipokines induced by obesity may directly impact Sertoli cells, causing chronic inflammation and oxidative stress, which in turn reduce the synthesis of inhibin B (22). Changes in the local testicular microenvironment, such as increased scrotal temperature and reduced blood supply due to abdominal obesity, may also negatively affect the interaction between Sertoli cells and germ cells (23).

Our study results indicated that the association between BMI and AMH/INHB did not exhibit significant interaction effects across all covariate subgroups, suggesting that this relationship was relatively independent of confounding factors. In all variable subgroups, except for Other Hispanic and other race, BMI showed a consistent negative correlation trend with AMH/INHB, with some subgroups exhibiting statistical significance. In contrast, Other Hispanic and other race exhibited a positive correlation with a larger effect size. This was illustrated in the forest plot, where the confidence intervals were quite wide, reflecting result instability, possibly related to small sample sizes and high data variability. Thus, caution was warranted in interpreting these findings.

To enhance the representativeness and reliability of our findings, we utilized data from NHANES, which employed a multi-stage random sampling methodology. A weighted analysis was conducted to adjust for sampling design and non-response, further strengthening the validity of the results. However, several limitations should be noted. First, the assessment of AMH and INHB was conducted over three cycles and involved specific subsamples, resulting in a limited sample size of 728 cases. The cleaning of invalid data and handling of missing values may have introduced information loss and selection bias. Additionally, the cross-sectional design restricts causal inferences, and the selection of confounding factors was not exhaustive, as FSH and physical activity were not considered.

## 5 Conclusion

Our study investigated the impact of BMI on serum levels of AMH and INHB in men, revealing that BMI is associated with a relatively independent negative correlation with these hormones. This relationship is more pronounced in obese individuals and shows no significant interaction with other confounding factors. The results indicate that higher BMI is significantly correlated with lower levels of both AMH and INHB. However, due to the low effect size of BMI on AMH, caution is warranted in interpreting its clinical significance. While we identified a non-linear relationship and key thresholds between these variables, further research with larger sample sizes is necessary to validate these findings and to better understand the complexities involved.

## Data Availability

The original contributions presented in the study are included in the article/**Supplementary Material**. Further inquiries can be directed to the corresponding author.
